# Kaposi sarcoma of the eyelid

**DOI:** 10.11604/pamj.2018.29.182.9651

**Published:** 2018-03-28

**Authors:** Fouad Chraibi, Idriss Andaloussi Benatiya

**Affiliations:** 1 University Allal Ben Abdellah, University Hospital Hassan II, Fez, Morocco

**Keywords:** kaposi sarcoma, eyelid, surgical excision

## Image in medicine

Kaposi's Sarcoma is a general angiosarcoma induced by viral growth factors, including interleukin 6 of Human Herpes Virus Type 8 ( HHV-8 ). It is preferably localized at limbs extremeties. The eyelid localization is very rare. This is an old patient of 68 years, which has since 3 years superior eyelid swelling of the right eye (A). Biopsy histopathological examination shows an aspect of Kaposi 's sarcoma. HIV serology was negative. The treatment then is surgical excision without safety margins with reconstruction in a the same operative time: external canthotomy and upper eyelid sliding flap sutured end-to-end. The outcome was favorable with a good cosmetic result without local recurrence after 6 months follow up (B).

**Figure 1:**
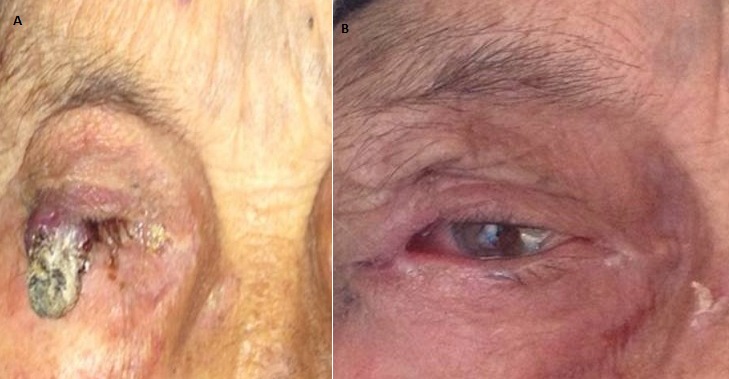
A) kaposi sarcoma: before surgery; B) kaposi sarcoma: after surgery

